# Trends and determinants of pregnancy loss in eastern Ethiopia from 2008 to 2019: analysis of health and demographic surveillance data

**DOI:** 10.1186/s12884-022-04994-4

**Published:** 2022-08-31

**Authors:** Lemma Demissie Regassa, Assefa Tola, Gamachis Daraje, Merga Dheresa

**Affiliations:** 1 Department of Epidemiology and Biostatistics, College of Health and Medical Sciences, Haramaya University, Harar, Ethiopia; 2 Department of Statistics, College of Computing and Informatics, Haramaya University, Haramaya, Ethiopia; 3 School of Nursing and Midwifery, College of Health and Medical Sciences, Haramaya University, Harar, Ethiopia

**Keywords:** Pregnancy loss, Stillbirth, Abortion, Miscarriage, Pregnancy outcome, Kersa HDSS, Ethiopia

## Abstract

**Background:**

Pregnancy losses remain a neglected issue and it will be taking more than a century before a pregnant woman in Sub Sahara has the same chance of her baby being born alive as a woman in a high-income country. Pregnancy loss data are limited and not universal in Sub Saharan countries. This study was aimed to assess the magnitude and determinants of pregnancy loss in eastern Ethiopia.

**Methods:**

This study was conducted in, open continues and dynamic cohort of population, Kersa Health and Demographic Survillance site (HDSS) in Eastern Ethiopia in 2008–2019. All mothers who had known pregnancy outcomes during the period and reside in Kersa HDSS were considered. The prevalence proportions were calculated as the sum of all pregnancy loss divided by the number births in the specified year. Log-Binomial regression was used to determine factors associated with pregnancy loss. Prevalence Proportion Ratio (PPR) was used to report the magnitude and strength of association. A p-value of less than 0.05 was considered statistically significant.

**Results:**

From 39,153 included pregnancies, 810 (20.7; 95%CI:19.32, 22.15 per 1000 births) experienced pregnancy loss. Stillbirth was higher than abortion (11.14 Vs. 9.55 per 1000 births). Lacking own income (aPPR:1.26; 95%CI: 1.01, 1.58), being daily laborer (aPPR:1.44; 95%:1.08, 306) history of previous pregnancy loss (aPPR:2.26, 95%CI:1.69, 3.03), unwanted pregnancy (aPPR:1.26; 95%CI:1.01, 1.80), not receiving antenatal care (aPPR:1.59; 95%CI: 1.19, 2.13) and not receive the TT-vaccine during pregnancy (aPPR:1.33; 95%CI: 1.08, 1.80) were positively associated with pregnancy loss.

**Conclusions:**

The overall rate pregnancy loss was ranged between 19.32, 22.15 per 1000 births with higher still births than miscarriage or abortion. Pregnancy loss was positively associated with social factors reproductive health factors, and maternal health service utilization.

## Background

Pregnancy losses majorly include preterm birth, stillbirth, and low birth weight, which are the major cause of neonatal morbidity, mortality and long-term physical and psychological problems [1]. Pregnancy losses remain a neglected issue, invisible in policies and programs, underfinanced and in urgent need of attention [2]. Worldwide in 2015, for every 1000 total births, 18.4 babies were stillborn, mostly in low- and middle-income countries, and 160 years will pass before a pregnant woman in Africa has the same chance of her baby being born alive as a woman in a high-income country today [3–5].

These Adverse birth outcomes; prematurity, low birth weight, and stillbirth represent significant problems in both developing and developed countries [6]. Each year, about 15 million babies in the world, more than one in 10 births, are born too prematurely [6, 7]. More than one million of those babies die shortly after birth; countless others suffer from lifelong physical, neurological, or educational disabilities, often at great cost to families and society [8, 9].

Among the 136 million babies born every year, approximately 2.6 million are stillborn. In 2006, 12% of babies are born prematurely, 8% with low birth weight, and 3% have major birth defects globally [10]. Recent report by UN indicated one stillbirth occurs every 16 s, and vast majority of stillbirths, 84%, occur in low- and lower-middle-income countries [11]. This report also indicated over 40& of stillbirths occur during labour. Therefore trends of pregnancy loss in one country could be the indicator for accessibility to maternal health care, and availability of professional birth assistances.

Pregnancy loss is global agenda as universal access to Sexual and Reproductive Health by 2030 is part of the Sustainable Development Goals [12]. In countries like Ethiopia where the preventive program “preconception care” has not yet been implemented, quantifying pregnancy loss and its predictors can help lay the groundwork for its introduction.

Several low-and middle-income countries (LMIC) use Demographic and Health Surveys and/or Health and Demographic Surveillance System to monitor the health of their population. From this surveillance, the regional disparities of pregnancy loss are evident, as the Sub-Sahara African region alone bears 66% of the burden [1, 10, 13].

Wide discrepancies were seen among previous reports on determinants of pregnancy loss and their importance in other Sub-Saharan African countries [14–17]. Meanwhile there are few studies focusing on the changes of incidence over the long period. Despite of requirement of evidence to show the trends and predictors of pregnancy loss there is scarce of published facts on the trends of pregnancy loss. We aimed to assess magnitude and determinants of pregnancy loss in eastern Ethiopia, from January1, 2008 to December 31, 2019.

## Methods

### Study area

**Fig. 1:**
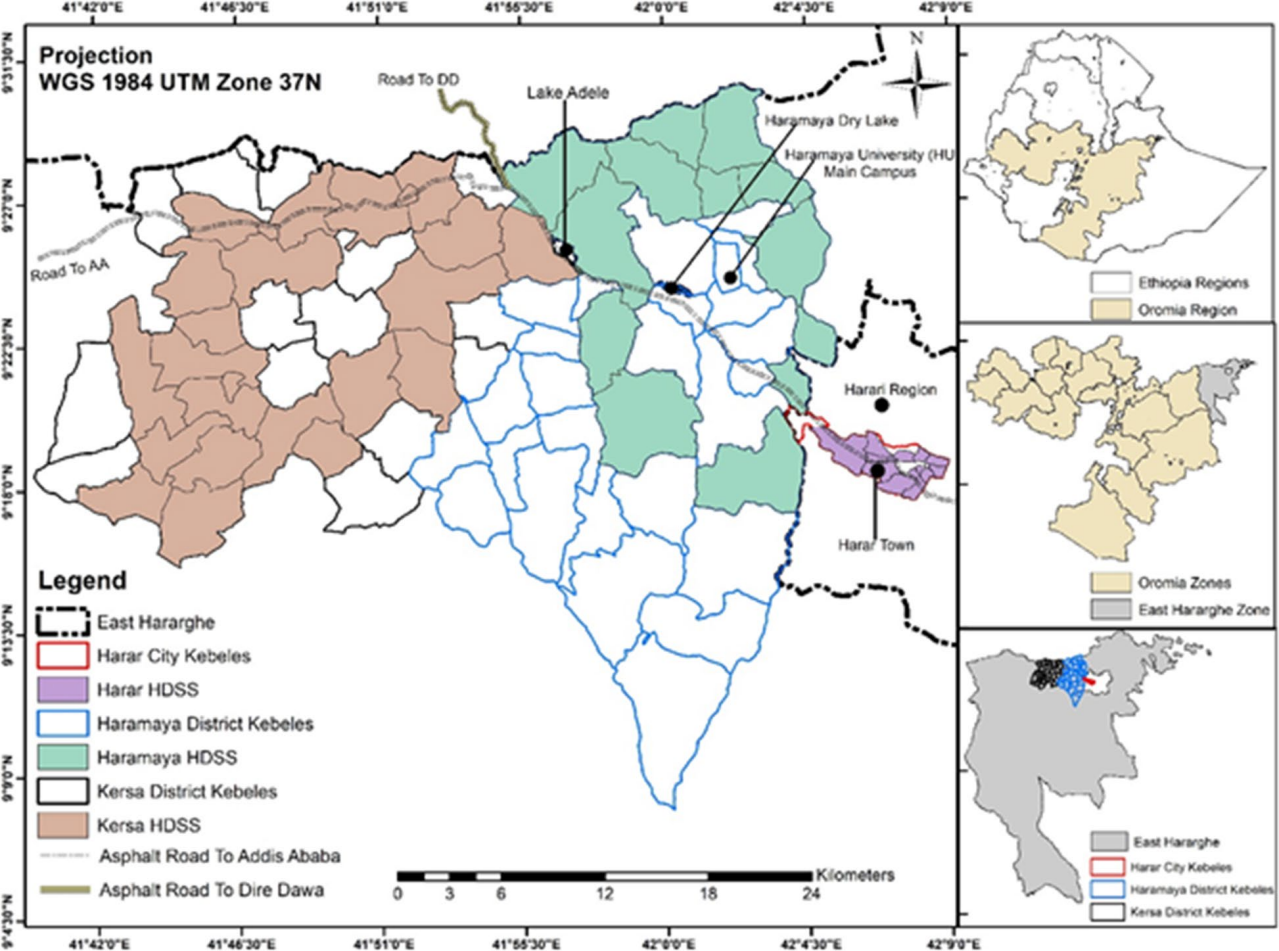
Map of Kersa HDSS sketched in 2019

It was conducted in the Kersa Health and Demographic Surveillance Site (HDSS) in Eastern Ethiopia from January, 2008 to December 2019. Kersa HDSS is an open continues and dynamic cohort of population established in 2007. The surveillance started after conducting a baseline census in 2007 and followed by population update and event registration with house-to-house visits every 6 months. The catchment was established in 12 sub-districts of Hararghe district, Eastern Hararghe, Oromia Region, Ethiopia. The study site expanded to Harar town and encompasses 6 kebel in 2013. Both site (Kersa site and Harar site) doubled thier study kebles to 24 and 12 respectivlily in 2015. Currently Kersa HDSS is operiting on 36 Kebeles with 40,310 house holde. At the end of 2015, the population was 116,325. Until the end of 2015, 217,819 births and 4,475 deaths were registered respectivily. Over 85% of births and deaths occurred at home. The annual net population growth ranges from -0,1 to 1.6. Meanwhile, the population growth rate ranged 1.63 to 2.94. The Total Fertility Rate ranges from 3.5 to 5.3 [18]. Khat, fruits and vegetables are important cash crops. Coffee is also an important cash crop; over 50 square kilometres are planted with this crop [19] (Fig. 1).

### Study design and period

An open dynamic cohort study design that longitudinally follow a well-defined entity of primary subjects (individuals, households, and residential units) and all related socio-demographic and health-related outcomes within a clearly defined geographic area. We included event records (data) of women whose outcome of pregnancy was registered from January 2008 to December 2019.

### Population and selection criteria

All mothers who had pregnancy outcomes during the period of January 2008 to December 2019 and reside in Kersa HDSS were considered as the study population. Data with incomplete with outcome variable were excluded from the study.

### Sample size and sampling procedure

Researchers extracted 12 years (January 1, 2008- December 30, 2019) data from Kersa HDSS database system. All households with mothers who had ever pregnant and had pregnancy outcome during the study period were considered for health and demographic surveillance were considered. We extracted data of 39,153 women from the Kersa HDSS database for this analysis.

### Data collection methods

Data were collected by well-trained regular Kersa HDSS staffs through face to face interview using tablet computer with Open Data Kit (ODK) collect application. The data collectors update the list of individuals living in the house during the 6-monthly field visits, by recording births, deaths and in- or out-migration. Changes in marital status through marriage, divorce, death of husband or wife or other separation are also recorded. Women are asked whether they were pregnant and about the pregnancy outcome. The economic status of individuals is updated every 2 years and assessed in detail every 5 years. In addition to death registration for the deceased, verbal autopsies are taken from close relatives, typically after a mourning period of 45 days. Supervisors were assigned to supervise data collectors in the field. Field supervisors checked data quality before it was sent to the database system. If supervisors found a data quality problem, it sent back to data collectors for correction. Collected data using a tablet computer in the field was temporarily stored on ODK aggregate. The data manager approved the quality of data and migrated data from temporary storage to the final storage “Openhds” database system.

### Variables and measurements

#### Outcome variable

Outcome variable of this study was pregnancy loss. Pregnancy losses are those pregnancy outcomes other than normal live birth which majorly includes preterm birth, stillbirth, and low birth weight which are the major cause of neonatal morbidity, mortality and long-term physical and psychological problems. In our study, pregnancy loss is referred to abortion, miscarriage or stillbirth. Both abortion and stillbirth were defined using a modified WHO criterion of fetal death [20, 21]. Abortion was defined as the deliberate termination of a human pregnancy, most often performed during the first 28 weeks of pregnancy. A miscarriage, or spontaneous abortion, is an event that results in the loss of a fetus before 28 weeks of pregnancy. On the other hand, stillbirth was defined as the birth of a baby who has died any time from 28 weeks of pregnancy through to the due date of birth. The baby may have died during the pregnancy or during birth. For analysis the pregnancy loss was classified as early (Miscarriage or abortion) and late pregnancy loss (still birth) [22].

#### Independent variables

We included sociodemographic (like age at first birth, level of literacy, occupation, household income, and wealth index), fertility history (like number of pregnancies, number of births, pregnancy wanted, experience of previous pregnancy) and health care utilization history (like antenatal care and TT-vaccination) to assess pregnancy loss.

### Statistical analysis

Data were described using means and standard deviations for continuous variables and rates and percentages for categorical data. Using an adaptation of the Delphi method, we developed shortened wealth indices (reclassified to three categories using the Kappa value). First, we recoded all categorical variables to binary variables. For questions with multiple response options, we recoded each response option as a binary variable (none were merged together). We removed response options with zero cases, as well as those common variables that were not included in the country-specific questionnaire. We then conducted a principal component analysis on all variables, with responses weighted at the individual level, and created a score from the factor weights of the first principal component. Scores were ordered and respondents were divided into 5 equal quintiles. Then we reclassified the quartiles in the lowest 2 quintiles, the middle quintile, and the highest 2 quintiles. The reclassification is deemed more programmatically meaningful, reliability as previous studies indicated [23].

The prevalence proportions were calculated as the sum of all pregnancy loss divided by the number of births in the specified year. The average annual percentage change in pregnancy loss (abortion and still birth) was calculated as [(a/b)(1/r) -1] × 1000, where a defines the most recent (2019) pregnancy loss rate, b defines the earliest (2007) pregnancy loss rate and r defines the number of years, which for this analysis is 12 years. We summarize the trends of pregnancy loss with its 95% confidence interval. To determine the factors associated with pregnancy loss, we used the cumulative data for the years 2008 to 2019. Multivariate analysis using Log-Binomial regression was used to determine factors associated with pregnancy loss (stillbirth and abortion or miscarriage). The Log Binomial regression approach models the probability of having the outcome (pregnancy loss) based on the binomial distribution and the logarithm of the probability as the link function in a generalized linear model [24, 25]. Log Binomial regression was chosen over Cox and robust Poisson regressions for three reasons. First the Cox regression produced standard errors that were too large, whereas the log-binomial model and the robust Poisson model had the correct type I error probabilities. Second, robust Poisson regression overestimate the parameters, whereas it was not happened with the log-binomial model. Third, the Akaike information criteria (AIC) was minimum in Log Binomial regression. We used backward elimination method to select variables; include variables with p-value less than 0.2 in binary negative binomial regression into final model. Model goodness of fit was checked by “countfit” of stata package. Count fit of stata package is used to compare count model and includes prediction value, person chi square (for goodness of fit test) and other information criteria (AIC and BIC) [26]. Prevalence Proportion Ratio (PPR) was used to report the magnitude and strength of association. In this study reporting PPR was deemed more appropriate than reporting odds ratio (OR) due to considerable “overestimation” of the strength of the association by OR [27, 28]. A p-value of less than 0.05 was considered statistically significant. All data were analyzed using STATA v.16.0.

## Results

### Participant’s characteristics

Data of 39,153 women who had pregnancy outcomes since January 2008 and before January 2020 were employed for data analysis. Median (IQR) age of women was 27.87 (9.94) years and range from 13.13 to 48.96 years. Majority of the respondents were Oromo (89.85%) and Muslims (91.03%). Regarding educational level, more than half (63.96%) of the women neither read nor write and 34.19% were literates. Of the total, 30,636 (78.25%) were housewives and only 1,403 (3.58%) were employed. More than half (66.65%) of the respondents have their own income and 33.68% were found in first quintile of wealth index (Table 1).

**Table 1:** Characteristics of women who ever gave birth between 2008 and 2019 in Kersa DHS site, eastern Ethiopia

Variables	Frequency	Percentage
**Ethnicity**		
Oromo	35,171	89.85
Amhara	2,521	6.44
Somali	80	0.20
Gurage	671	1.71
Harari	501	1.28
Tigre	90	0.23
Other	112	0.29
**Religion**		
Muslim	35,642	91.03
Orthodox Christians	3,121	7.97
Others	390	0.99
**Education**		
Formal education	13,350	34.19
Read only	216	0.55
Read and write	509	1.30
Can’t read and write	24,976	63.96
**Occupation**		
Housewife	30,636	78.25
Student	2,506	6.40
Unemployed	1,922	4.91
Employee	1,403	3.58
Farmer	1,013	2.59
Trader	878	2.24
Daily laborer	502	1.28
Others	293	0.75
**Have own income**		
Yes	26,097	66.65
No	13,056	33.35
**Wealth Index**		
First quintile	10,847	33.68
Second quintile	11,987	33.49
Third quintile	12,506	34.94
**Number of pregnancies**		
One	7,758	19.81
Two -Five	22,473	57.40
Six -Ten	8,118	20.73
More than 10	804	2.050
**Preceding child alive**		
Yes	30,530	77.98
No	865	2.21
First pregnancy	7,758	19.81
**Number of alive children**		
One	6,812	17.40
Two -Five	22,619	57.77
Six -Ten	7,795	19.91
More than 10	1,927	4.92

Median (IQR) age at first birth was 18.52 (2) years. The minimum and maximum age at first birth was 13 and 30 respectively. For women who gave birth previously, 30,533 (77.98%) preceding children was alive while 865 (2.21%) died. Mean (± SD) number of pregnancies was 3.72 (2.36), 7,758 (19.88%) were first pregnancy. Mean (± SD) number of alive births was 2.17 (0.87) per women. During pregnancy, only 12,437 (31.77%) women were received antenatal care and 2,743 (35.65%) received tetanus toxoid vaccine. Participants characterized is summarized in Table 1.

### Trends of pregnancy loss

**Fig. 2:**
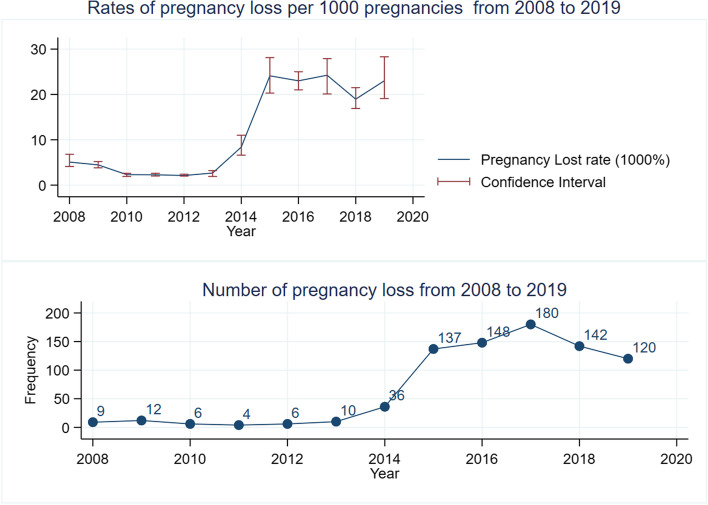
Trends of pregnancy lost by record years in Kers HDSS in Eastern Ethiopia from 2008 to 2019. Legends: **a**: number of pregnancy loss from 2008 to 2019; (**b**): rate of pregnancy loss per 1000 from 2008 to 2019

From 39,153 pregnancies from 2008 to 2019, 436 (1.11%) resulted in still birth and 374 (0.96%) miscarriage or abortion. A larger number of births (5,591/39,153), abortion (83/384), and stillbirth (97/436) were recorded in 2017. Total still birth and abortion rates were 11.14 per 1000 births (95%CI: 10.14, 12.22) and 9.55 per 1000 births (95%CI: 8.64, 10.57) respectively. The percentage of pregnancy loss was increasing over the years and the highest pregnancy loss was recorded in 2015 (Fig. 2).

The average annual decline rate in the overall pregnancy loss rate was 4.10%. Still birth takes the highest size of pregnancy loss and responsible for the increased trend of pregnancy loss. Overall, the rate of pregnancy loss was 20.7 per 1000 births (20.69, 95%CI:19.32, 22.15). Pregnancy loss was higher among mothers who did not receive antenatal care and not receive tetanus toxoid vaccine. Proportion of pregnancy loss was higher among women whose occupation was a daily laborer. Both abortion and stillbirth outcome were lower among women with formal education, daily laborer and women from household with poor wealth quintile (Table 2).

**Table 2:** Rate of pregnancy loss among women who gave birth during 2008- 2019 by different characteristics of the women

Variables	Pregnancy loss		Pregnancy loss rates (per 1000)		Chi.2
	Yes	No	Early PL	Late PL	
**Education**					0.73
Neither read nor write	524	24452	5.46 (4.63, 6.45)	6.68 (5.69, 7.84)	
Formal education	271	13079	5.09 (4.01, 6.46)	7.81 (6.31, 9.67)	
Read or write	13	712	6.78 (2.83, 16.19)	2.71 (0.68, 10.78)	
**Occupation**					20.76*
Housewife or farmer	675	30,974	5.80 (5.03, 6.70)	7.05 (6. 11, 8.12)	
Employed	16	1387	2.77 (1.04, 7.37)	4.85 (2.31, 10.14)	
Unemployed	84	4344	3.59 (2. 20, 5.85)	6.28 (4.34, 9.09)	
Daily laborer	13	489	3.9 (0.10, 15.65)	13.75 (5.94, 31.52)	
Trader	9	869	1.09 (0.15, 7.70)	5.43 (1.93, 15.25)	
Others^+^	13	220	13.56 (5.07, 35.77)	16.95 (7.03, 40.28)	
**Has own income**					9.03*
Yes	500	25,597	9.08 (8.00, 10.32)	10.078 (8.94, 11.36)	
No	310	12,746	10.49 (8.88, 12.39)	13.25 (11.43, 15.36)	
**Wealth Index**					9.52*
First quintile	281	10,566	12.45 (10.52, 14.71)	13.46 (11.46, 15.81)	
Middle quintile	236	10,979	10.25 (8.55, 12.30)	10.79 (9.04, 12.88)	
Third quintile	209	9,934	9.07 (7.40, 11.11)	11.54 (9.63, 13.81)	
**Preceding Child alive**					55.64*
Yes	526	30,007	8.45 (7.48, 9.54)	8.78 (7.79, 9.89)	
No	220	5830	19.49 (15.256, 24.87)	30.32 (24.94, 36.83)	
First pregnancy	64	2,506	10.51 (7.21, 15.28)	14.39 (10.45, 19.81)	
**Number pregnancy**					7.63
One	188	7,570	9.41 (7.49, 11.82)	14.82 (12.36. 17.77)	
Two -Five	441	22,032	9.70 (8.49, 11.07)	9.92 (8.71, 11.31)	
Six -Ten	166	7,952	9.24 (7.37, 11.57)	11.21 (9.14, 13.75)	
More than 10	15	789	9.95 (4.98, 19.78)	8.71 (4.15, 18.15)	
**ANC**					64.72*
Yes	197	12,240	4.18 (3.19, 5.48)	11.66 (9.92, 13.70)	
No	613	26,103	12.05 (10.81, 13.43)	10.89 (9.72, 12.21)	
**Vaccinated TT- during**					67.42*
Yes	93	8,071	3.67 (2.57, 5.25)	7.72 (6.03, 9.87)	
No	717	30,272	11.10 (9.99, 12.33)	12.04 (10.88, 13.31)	

+ : others include students, retired, *PL*: Pregnancy loss, *Chi.2* Chi-square value

*: *p*-value < 0.05

### Predictors of pregnancy outcome

In Log-Binomial multivariate, pregnancy loss was significantly associated with having one’s own income, occupation, previous pregnancy experience, pregnancy wantedness, receiving antenatal care and receiving TT-vaccine (Table 3).

**Table 3:** Factors associated with pregnancy loss in Kersa HDSS from 2007 to 2019, Eastern Ethiopia

Pregnancy loss	No (%)	Yes (%)	cPPR (95%CI)	aPPR (95%CI)
**Education**				
Have formal education	13079 (97.97)	271 (2.03)	1	1
Read or write	712 (98.21)	13 (1.79)	0.88 (0.51, 1.53)	1.11 (0.52, 2.37)
Can't read and write	24452 (97.9)	524 (2.10)	1.03 (0.89, 1.20)	0.99 (0.78, 1.26)
**Having own income**				
Yes	25597 (98.08)	500 (1.92)	1	1
No	12746 (97.63)	310 (2.37)	1.24 (1.08, 1.43)	1.26 (1.01, 1.58) *
**Wealth index**				
Poor	17428 (97.94)	367 (2.06)	1	1
Middle	10981 (97.91)	234 (2.09)	1.01 (0.86, 1.19)	1.18 (0.91, 1.53)
Rich	9934 (97.94)	209 (2.06)	1.00 (0.84, 1.18)	1.20 (0.91, 1.57)
**Occupation**				
Housewife	30974 (97.87)	675 (2.13)	1	1
Employee	2534 (98.45)	40 (1.55)	0.73 (0.53, 1.00)	0.78 (0.46, 1.32)
Unemployed	4346 (98.15)	82 (1.85)	0.87 (0.69, 1.09)	0.83 (0.59, 1.17)
Daily laborer	489 (97.41)	13 (2.59)	1.21 (0.71, 2.09)	1.44 (1.08, 3.06) *
**Age at first birth**				
Under 18 years	8697 (98.3)	150 (1.7)	1	1
18–25 years	27257 (97.82)	608 (2.18)	1.29 (1.08, 1.54)	1.06 (0.81, 1.39)
> 25 years	2389 (97.87)	52 (2.13)	1.26 (0.92, 1.72)	1.16 (0.69, 1.93)
**Previous birth alive**				
Yes	32766 (98.25)	585 (1.75)	1	1
No	3071 (95.02)	161 (4.98)	2.84 (2.39, 3.37)	2.26 (1.69, 3.03) **
First Pregnancy	2506 (97.51)	64 (2.49)	1.42 (1.10, 1.83)	0.81 (0.52, 1.26)
**Pregnancy wanted**				
Yes	10,716 (98.11)	206 (1.89)	1	1
No	4052 (96.87)	131 (3.13)	1.66(1.34, 2.06)	1.26 (1.01, 1.58) *
**Number of pregnancies**				
One	22,000 (97.9)	473 (2.1)	1	1
two -five	7973 (98.21)	145 (1.79)	0.92 (0.78, 1.09)	0.97 (0.73, 1.30)
Six-ten	789 (98.13)	15 (1.87)	0.78 (0.63, 0.97)	0.95 (0.66, 1.37)
More than ten	22,000 (97.9)	473 (2.1)	0.82 (0.49, 1.38)	0.89 (0.36, 2.22)
**Receive TT vaccine**				
Yes	8055 (98.66)	109 (1.34)	1	1
No	30288 (97.74)	701 (2.26)	1.69(1.39, 2.07)	1.33 (1.08, 1.80) *
**Received antenatal care**				
Yes	12212 (98.19)	225 (1.81)	1	1
No	26131 (97.81)	585 (2.19)	1.21(1.04, 1.41)	1.59 (1.19, 2.13) **

*aPPR* Adjusted prevalence proportion ratio, *cPPR* Crude prevalence proportion ratio, *CI* Confidence Interval, *TT* Tetanus Toxoid

Women who did not had their own income were associated with a higher pregnancy loss (aPPR: 1.26; 95%CI: 1.01, 1.58) compared to mothers who have their own income. Compared to housewives, the proportion of pregnancy loss was higher by 44% (aPPR: 1.44; 95%: 1.08, 306) among daily laborer women. The proportion of pregnancy loss was more than two (aPPR:2.26, 95%CI: 1.69, 3.03) times higher among women whose preceding child was not alive. On the other hand, the proportion of pregnancy loss was higher by 26% (aPPR: 1.26; 95%CI: 1.01, 1.80) among women with unwanted pregnancy than that of wanted pregnancy. Compared to women who attend antenatal care, the proportion of pregnancy loss was higher by 59% (aPPR:1.59; 95%CI: 1.19, 2.13) among women who did not receive antenatal care. Similarly, women who did not receive the TT-vaccine during her pregnancy was associated with a higher pregnancy loss rate (aPPR:1.33; 95%CI: 1.08, 1.80) (Table 3).

## Discussion

The overall rate pregnancy loss was just above 20 per 1000 births and sharply increased between 2012 and 2015 then became irregular up to 2019. Pregnancy loss was absolutely related to social factors (including haven’t own financial income and being daily laborer), and pregnancy experience (including unwanted pregnancy, previous pregnancy not alive). However negatively associated with prenatal services (ANC, and TT vaccine) exposure of the mothers.

Pregnancy loss was more common among women without their own sources of income. Previous study conducted in Republic of Korea indicated the low income level is related to any negative pregnancy outcome [29]. Mothers who generate self-income are reported to have more power to make decision on family planning, and other maternal health service utilization [30].

Women who worked as daily laborers had a greater odd of pregnancy loss than housewives. Although it is difficult to draw strong conclusions, because they tend to be retrospective and confounded by alternatives, occupation can affect the pregnancy in different ways. One reason of pregnancy loss among daily laborer might be from occupational and environmental exposures like smoking, alcohol, and caffeine-have [31, 32]. The other cause might occupational hazardous or accidents as daily laborer are expected to lift heavy materials. Studies strengthen this evidence by reporting daily laborer women are working marginal, casual jobs with little regulatory protection, they hustle from engagement to engagement, typically for as little pay or as many hours the boss wants [33, 34]. Hence the long working hours and physical exertion, likely affect obstetric outcomes.

Prior pregnancy loss appears to increase the likelihood of subsequent pregnancy loss. This suggests a potentially inheritable component. Review conducted in 2009 reported significant number of pregnancy loss was associated with a parental balanced structural chromosome rearrangement, chromosomal inversions, insertions, and mosaicism [35]. The repeated pregnancy loss could be due to anatomical [36], environmental [31], endocrine [31], thrombotic [37] or distress and behavioral [38] etiologies.

Not attending antenatal care and not receiving TT-vaccine remained associated factors for pregnancy loss. Different studies evident antenatal care decrease pregnancy and birth complications by increasing the access to essential micronutrient supplementation, screening and treatment for complications, immunization against tetanus and insecticide-treated mosquito nets to help prevent this debilitating and sometimes deadly disease [39, 40].

This research has a number of advantages over previously reported results in Ethiopia. The first advantage is that we used the large surveillance data which was collected over wide range of years. This depicts the prevalence of pregnancy loss in the area. The model used to determine predictors is the second strength. By avoiding overestimation, the Log Binomial regression reduces the number of voices in the data. Our research, on the other hand, is not without limitation. Possible clinical risk factors like uterine or cervical problems, diabetes, hypertension disorder and epilepsy were not included in this study’s findings. Behavioral factors including obesity, smoking, chewing Khat, alcohol, illicit drugs and medication were not included in this analysis. Despite these limitations, we included socioeconomic and health-care data, as well as the trajectory of pregnancy loss. As a result, the results should be viewed with those limitations in mind.

## Conclusion

Inconclusion pregnancy loss was increasing over the last five years (2015–19) and positively associated with low own low socioeconomic status and adverse pregnancy experience. But negatively associated with antenatal services (ANC, and TT vaccine) exposure of the mothers.

Therefore, to improve pregnancy outcomes in low-socioeconomic women, fundamental support to receive more prenatal care or to modify lifestyle risk factors, such as long working hours, may be needed in addition to lowering the problem of pregnancy loss. This finding suggests that the health policy and programs should not solely concentrate on the enhancement of adequate prenatal care. Instead, there is a need for social interventions aimed at more in-depth and distal determinants of health to improve pregnancy outcomes in pregnant women.

## Data Availability

The data that support the findings of this study are available from Hararghe Health and Demographic Surveillance office, but restrictions apply to the availability of these data, which were used under license for the current study, and so are not publicly available. Data are however available from the authors upon reasonable request and with permission of Hararghe Health and Demographic Surveillance office (mderesa@yahoo.com).

## Abbreviations

AIC: Akaike information criteria
ANC: Antennal care
TT: Tetanus Toxoid
HDSS: Kers Health and Demographic Health Surveillance Site
LMIC: Low-and Middle-income countries
PPR: Prevalence Proportion Ratio
IRB: Institutional Review Board
IQR: Inter quartile range
SD: Standard deviation
